# Biological function and clinical relevance of chromogranin A and derived peptides

**DOI:** 10.1530/EC-14-0027

**Published:** 2014-04-29

**Authors:** Maria Angela D'amico, Barbara Ghinassi, Pascal Izzicupo, Lamberto Manzoli, A Di Baldassarre

**Affiliations:** Section of Human MorphologyDepartment of Medicine and Aging Sciences, G. d'Annunzio University of Chieti–Pescara, Via Dei Vestini 31, 66013 Chieti, Italy

**Keywords:** Chromogranin A, Vasostatin, Pancreastatin, Parastatin

## Abstract

Chromogranin A (CgA (CHGA)) is the major soluble protein co-stored and co-released with catecholamines and can function as a pro-hormone by giving rise to several bioactive peptides. This review summarizes the physiological functions, the pathogenic implications, and the recent use of these molecules as biomarkers in several pathological conditions. A thorough literature review of the electronic healthcare databases MEDLINE, from January 1985 to September 2013, was conducted to identify articles and studies concerned with CgA and its processing. The search strategies utilized keywords such as chromogranin A, vasostatins 1 and 2, chromofungin, chromacin, pancreastatin, catestatin, WE14, chromostatin, GE25, parastatin, and serpinin and was supplemented by the screening of references from included papers and review articles. A total of 209 English-language, peer-reviewed original articles or reviews were examined. The analysis of the retrospective literature suggested that CgA and its several bioactive fragments exert a broad spectrum of regulatory activities by influencing the endocrine, the cardiovascular, and the immune systems and by affecting the glucose or calcium homeostasis. As some peptides exert similar effects, but others elicit opposite responses, the regulation of the CgA processing is critical to maintain homeostasis, whereas an unbalanced production of peptides that exert opposing effects can have a pathogenic role in several diseases. These clinical implications entail that CgA and its derived peptides are now used as diagnostic and prognostic markers or to monitor the response to pharmacological intervention not only in endocrine tumors, but also in cardiovascular, inflammatory, and neuropsychiatric diseases.

## Introduction

Granin family includes a group of acidic soluble proteins expressed by a variety of endocrine, neuroendocrine, and neuronal cells. They are co-stored in secretory granules and co-released with resident peptide hormones, neurotransmitters, or amines in response to a variety of physiological and pharmacological stimuli. Nine members of the granin family have been presently described: chromogranins, namely chromogranins A (CgA) and B (CgB), and secretogranins, namely SgII, *1B1075* gene product (SgIII (*Scg3*)), HISL-19 antigen (SgIV), 7B2 (SgV), neuroendocrine secretory protein of *M*_r_ 55 000 (NESP55 or SgVI), VGF (SgVII), and proSAAS (SgVIII) (1). CgA, as well as the other granins, is characterized by i) an acidic pI due to high percentage of acidic amino acids (glutamic acid and aspartic acid), ii) heat stability due to its high hydrophilic nature, iii) the presence of multiple dibasic cleavage sites, and iv) the capacity to form aggregates and to bind calcium.

## Chromogranin A

CgA (CHGA) is an acidic protein with a molecular weight of 48 kDa that is composed of 439 amino acids and expressed by several normal or neoplastic cells of the diffuse endocrine and neuroendocrine systems or by some cancer cells that can undergo neuroendocrine differentiation. Its name is derived from the original discovery in adrenal medulla (2). CgA is co-stored and co-released with catecholamines from storage granules in the adrenal medulla, or with the parathyroid hormone in response to hypocalcemia in the parathyroid gland (3) (in this context, it is also referred as parathyroid secretory protein 1). It represents the most abundant protein among the phosphorylated proteins released by the parathyroid glands and its secretion and phosphorylation levels are inversely proportional to extracellular calcium concentration (4).

## Chromogranin A processing and derived peptides

**Figure 1:**
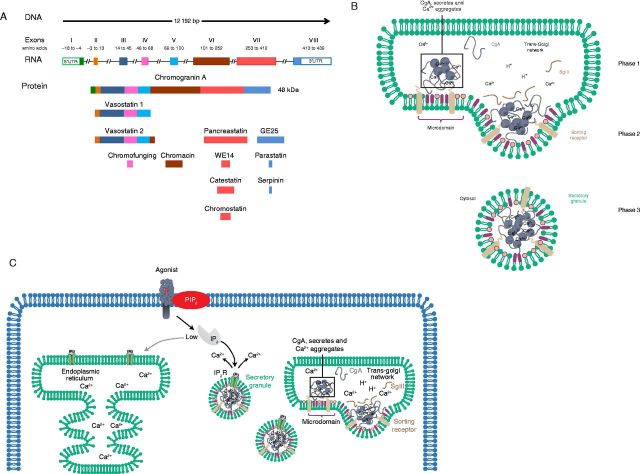
(A) Scheme of exonic regions of CgA and derived peptides. The human *CgA* gene is organized in eight exons and seven introns. The derived transcript is translated into the CgA protein with a molecular weight of 48–52 kDa. The proteolytic cleveages of specific CgA sequences by the pro-hormone convertases generate several bioactive peptides. Roman numerals indicate the exon sequences of the mRNA, whereas Arabic numerals identify the amino acids in the mature protein minus the signal peptide. (B) Schematic model of the role of CgA in the biogenesis of secretory granules. CgA aggregates with proteins co-stored into secretory granules in a Ca^2^^+^-dependent manner (phase 1); then, it interacts with SgIII present in the lipid-rich microdomains of the trans-Golgi network favoring the vesicle budding (phase 2); secretory granules are finally released into the cytosol (phase 3). C. IP_3_-dependent Ca^2^^+^ mobilization from intracellular stores. The binding of agonists (hormones and neurotransmitters) to the G-protein-coupled receptors determines the PIP_2_ hydrolysis and the inositol trisphosphate (IP_3_) generation. IP_3_ binds to specific receptors (IP_3_R), a family of Ca^2^^+^ channels responsible for the intracellular Ca^2^^+^ mobilization from intracellular stores, such as the endoplasmic reticulum (ER) or the secretory granules. The IP_3_R/Ca^2^^+^ channels present on the intracellular store membranes show different sensitivities and respond to different IP_3_ concentrations: low IP_3_ levels that are not sufficient to activate the IP_3_R of ER trigger the more sensitive IP_3_R/Ca^2^^+^ channels present on the membrane of secretory granules with the consequent release of Ca^2^^+^ from the granules into the cytoplasm.

The human *CgA* gene is located on chromosome 14q32.12, spans 12 192 bp, and is organized in eight exons and seven introns. The derived transcript of 2 kb is translated into the 457 residues CgA protein of ∼48–52 kDa molecular weight that undergoes post-translational processes and proteolytic cleavages by pro-hormone convertases. The CgA cleavages generate several biologically active peptides: vasostatins 1 and 2, chromofungin, chromacin, pancreastatin, catestatin, WE14, chromostatin, GE25, parastatin, and serpinin. The scheme depicted in Fig. 1A represents the exonic regions of *CgA* DNA sequence and the corresponding derived peptides. The 5′-UTR (259 bp) of the *CgA* mRNA and most of the signal peptide of CgA correspond to the exon I. The β-granin represents the highly conserved amino-terminal domain of CgA encoded by exons II–V and few amino acids encoded by the exon VI. It encompasses several bioactive peptides: vasostatin 1 (VST1: hCgA_1__–__76_) and vasostatin 2 (VST2: hCgA_1__–__113_) have vasorelaxant and cardiosuppressive properties. Chromofungin corresponds to the sequence Arg (47)–Leu (66) of the whole protein, interacts with the cell wall, crosses the plasma membrane, accumulates in the micro-organism, and inhibits calcineurin activity. Chromacin, the most variable across species, inhibits the growth of both Gram-positive and Gram-negative bacteria in bovines, and represents a general marker of neuroendocrine tumors (NETs) (5). Exon VII encodes for the dysglycemic hormone pancreastatin (PST: hCgA_250__–__301_), the catecholamine release-inhibitory and antihypertensive peptide catestatin, a 20 amino acid cleavage product that seems to be involved in pancreatic β-cell functions chromostatin, and the 14 amino acid peptide WE14 (hCgA_324__–__337_). The name of this molecule is derived from the presence of tryptophan (W) at the N-terminal position and of glutamic acid (E) at C-terminal position; it modulates histamine release from rat peritoneal mast cells and acts as auto-antigen in type 1 diabetes (6). Exon VIII encodes GE25, parastatin, and serpinin. GE25, whose bioactivity has not yet been determined, is expressed by the pituitary gland, gut, and pancreas. Parastatin corresponds to residues 347–419 of CgA and is secreted together with various sub-fragments by the parathyroid glands. It seems to be involved in a negative feedback loop, as it inhibits both parathyroid hormone and CgA secretion. Serpinin that corresponds to the C-terminal end of CgA (hCgA_403__–__428_) regulates granule biogenesis in endocrine and neuronal cells by inhibiting granule protein degradation in the Golgi complex and exerting a protective effect against oxidative stress. Serpinin's influence on cardiac activity has recently been reported (7).

Both pattern and rate of CgA processing vary in a tissue-specific manner. In adrenal medulla and anterior pituitary gland, rate and processing are low, while CgA is processed faster and more extensively in the endocrine pancreas and in gastrointestinal tissues. Proteolytic processing of CgA may also occur after its release from neuroendocrine cells.

## Physiological roles and clinical implications of CgA and its cleavage products

### CgA intracellular functions

#### Granule biogenesis

*In vitro* (8) and *in vivo* studies (9) demonstrated that CgA is the driving force for the biogenesis of secretory granules, because it aggregates in the acidic environment of the vesicles and induces the budding of the trans-Golgi network membranes forming dense-core granules. Moreover, CgA N-terminal region tightly binds the lipid-rich microdomains of trans-Golgi network membranes, thus influencing the pro-hormones transport into the secretory granules (the large dense-core vesicles) or, in the adrenal medulla, into the chromaffin granules (10) (Fig. 1B). CgA plays an important role also in replenishing the cells of secretory granules after the exocytosis. In particular, it seems to be up-regulating the biogenesis of dense-core granules through the serpinin-mediated inhibition of the degradation process.

#### Calcium homeostasis

CgA exerts a crucial role in calcium homeostasis, as it has high binding capacity but low affinity for Ca^2^^+^. The abundance (∼2–4 mM) of CgA inside the granules contributes to make dense-core granules the major intracellular calcium reservoir (11). At the same time, CgA properties facilitate the ready exchange of bound and free Ca^2^^+^ within secretory granules and the Ca^2^^+^ mobilization into the cytoplasm, through the activation of IP_3_R/Ca^2^^+^ channels that are present on the membranes of granules (Fig. 1C).

### CgA extracellular function

It is widely recognized that the adrenal medulla is the main source of circulating CgA, while adrenergic nerve endings and neuroendocrine cells secrete CgA in peripheral tissues. Present in the diffuse neuroendocrine system, it has also been detected in rat and human cardiac secretory granules where it is co-stored with natriuretic peptide hormones (12) and released mainly under stress conditions (13).

Even if logical and clinical evidences indicate a certain CgA involvement in the homeostasis control, a clear ‘endocrine role’ for CgA remains to be established.

Knockout mice for *CgA* expression are viable and fertile and do not show developmental abnormalities (9), even if they develop a severe hypertension (14). Their neural and endocrine functions are not grossly impaired and adrenal glands present regular structures with normal sizes and numbers of chromaffin cells. However, epinephrine, norepinephrine, and dopamine secretion rises significantly and the adrenal medullary expression of other dense-core secretory granule proteins including CgB (CHGB) and various secretogranins (SgII (SCG2)–SgVI (GNAS)) is up-regulated, suggesting that increased expression of other granins may compensate for the CgA deficiency (9). In humans, naturally occurring variation at the *CgA* gene contributes to alterations in autonomic function, and hence hypertension, as a consequence of changes in storage and release of CgA. It was reported that plasma CgA concentration positively correlates with catecholamine release rates and consequent blood pressure increase, probably for its essential role in granule size, number, density, and cargo storage regulation (14).

At the CNS level, CgA may play an autocrine role as a glucocorticoid-responsive inhibitor regulating the secretion of peptides derived from proopiomelanocortin in the pituitary gland (15). Moreover, CgA indirectly causes neuronal apoptosis by inducing microglial cells to produce both heat-stable diffusible neurotoxic agents and TNFα (16). Recent studies evidenced lower CgA (−44%) levels in amyotrophic lateral sclerosis patients compared with healthy individuals (17), whereas data on CgA involvement in psychiatric diseases are not univocal and studies on schizophrenic patients gave contradictory results (18, 19).

### CgA-derived peptides

**Figure 2:**
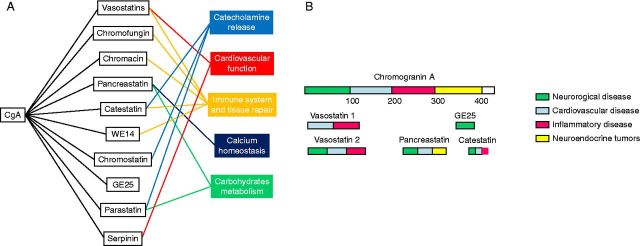
(A) Physiological effects of human CgA proteolytic fragments. The scheme summarizes the main physiological functions of CgA cleavage products. (B) CgA and its derived peptides as biomarkers. The scheme summarizes the current use of CgA and of the different cleavage products as biomarkers in neuroendocrine tumors and neurological, cardiovascular, and inflammatory diseases.

CgA can be cleaved into several bioactive fragments, which exert a broad spectrum of regulatory activities by influencing the endocrine, the cardiovascular, and the immune systems and by affecting the glucose or calcium homeostasis (Fig. 2A) (20). Some peptides exert similar effects, but others elicit opposite responses. For this reason, the regulation of the CgA processing in order to generate diverse molecules under different physiological conditions is critical for counterbalancing the effects and maintaining homeostasis.

#### Vasostatins 1 and 2

Vasostatins 1 (CgA_1__–__76_) and 2 (CgA_1__–__113_) represent the N-terminal fragments of CgA and exert a large spectrum of homeostatic actions, including vasodilation, antifungal and antimicrobial effects, modulation of cell adhesion, and inhibition of parathyroid hormone secretion. The CgA processing into vasostatin peptides occurs both at the cell membrane level and in the extracellular matrix (21). Vasostatins 1 and 2 are structurally very similar and induce comparable effects acting through autocrine, paracrine, and endocrine mechanisms (22). Their mechanisms of action are only partially elucidated. So far, classical, high-affinity receptors have not been identified, while receptor-independent cell penetration (e.g., antimicrobial action) or membrane perturbation (cardiac inotropism)-associated mechanisms have been postulated in endothelium and heart (23, 24). Vasostatins have been linked to vasculogenesis and remodeling (12). In contrast to catestatin, vasostatin inhibits VEGF-induced endothelial cell proliferation and migration and the formation of capillary-like structures (25). However, similar to catestatin, vasostatin has vasorelaxant properties and exerts negative inotropic and lusitropic effects on the heart, particularly in the presence of intense adrenergic stimuli. These cardiosuppressive effects (26) seem to be due to a non-competitive counteraction of the β-adrenergic-mediated positive inotropism (27). Together, the cardiotropic and vasoactive properties of vasostatins suggest that these peptides may play a role as homeostatic stabilizers of the cardiovascular system, particularly under conditions of sympathetic overstimulation, such as those occurring under stress response (22, 28).

In addition to cardiovascular effects, a regulatory role in the immune system has also been described. Recent studies have demonstrated that vasostatin modulates the innate immunity by inducing calcium entry into human neutrophils, an effect similar to that evoked by catestatin (29). Moreover, vasostatin directly inhibits growth of yeast, bacteria, and fungi by penetrating through their membranes. These effects are probably due to that part of the peptide that encompasses the chromofungin sequence.

Finally, vasostatins modulate pro-adhesive interaction of fibroblasts and smooth muscle cells with extracellular matrix proteins (30) and exert autocrine inhibition of parathyroid hormone secretion in the parathyroid cells (31).

#### Pancreastatin

Pancreastatin was the first identified CgA-derived peptide (32). The major form detected in human plasma consists of 52 amino acids (hCgA_250__–__301_) and requires C-terminal amidation to be active. Released with catecholamines from the sympathetic nervous system in stress situations, pancreastatin appears to be involved in the modulation of energy metabolism. Moreover, it influences multiple facets of both carbohydrate and lipid metabolism decreasing glucose uptake (by ∼50%) and increasing spillover of free fatty acids (by 4.5- to 6.4-fold) (33). This counter-regulatory function on insulin action can be directed to reinforce catecholamine action and extend its effect. In a situation of unbalanced sympathetic activation, an excess of catecholamines along with increased pancreastatin levels could contribute to the development of insulin resistance. This hypothesis is supported by the observation that pancreastatin levels rise in human hypertension and in gestational or type 2 diabetes. In addition to a direct dysglycemic effect, pancreastatin modifies the insulin:glucagon ratio stimulating glucagon and inhibiting insulin secretion stimulated by physiological activators (34). Nonetheless, the exact role of pancreastatin in the pathogenesis of the insulin-resistant states and diabetes remains to be elucidated.

The pancreastatin region of CgA gives rise to three genetic variants, one of which (Gly297Ser) substantially increases the peptide's potency to inhibit cellular glucose uptake. These observations suggest that hereditary alterations in pancreastatin's primary structure may give rise to interindividual differences in glucose and lipid metabolism.

Pancreastatin also inhibits pancreatic and gastric exocrine secretion and also the parathormone release.

#### Catestatin

Catestatin consists of a 21 amino acid peptide and acts at nicotinic cholinergic receptors as a potent autocrine inhibitor of catecholamine secretion. Targeted ablation of CgA locus in a mouse model results in severe hypertension that can be rescued by administration of the catestatin fragment. Moreover, patients with hypertension display increased CgA (35) and reduced catestatin plasma levels (36). These observations suggest that catestatin deficiency might play a role in the development of hypertension, whose pathogenesis has a significant neurogenic component based on a sustained overactivity of the sympathetic nervous system. Moreover, the individual genetic profile seems to influence the catestatin activity. In addition, the Gly364Ser genetic variant of catestatin seems to offer protection against the development of hypertension (37), whereas the CgA processing to catestatin appears to be more effective in women than in men (38).

Catestatin can induce cardiovascular responses at local as well as at systemic levels (39). In particular, it induces vasorelaxant and antihypertensive effects by means of the induction of histamine release from mast cells (40, 41). Catestatin also exhibits pronounced angiogenic and vasculogenic activities, as it induces migration and proliferation of endothelial cells and stimulates chemotaxis of vascular smooth muscle cells (42). Effects comparable to that of VEGF were identified *in vitro* in tube formation assays, as well as *in vivo* in the mouse cornea system (43, 44).

The catestatin involvement in inflammation has recently been highlighted in terms of chemotaxis and induction of pro-inflammatory cytokines (45, 46). These findings suggest a role in the neurodegenerative disease, as CgA represents an important constituent of the plaques in Alzheimer's disease (47) and the derived catestatin has a chemotactic effect on the monocytes that invade and surround the plaques (48). In addition, catestatin directly inhibits growth of fungi, yeast, and bacteria, including Gram-positive and Gram-negative, likely because of its highly cationic nature, a characteristic feature of the antibacterial compound (49).

#### Parastatin

Parastatin (CgA_347__–__419_) consists of a highly conserved CgA domain, described for the first time in the porcine parathyroid. Parastatin modulates parathormone release by porcine parathyroid cells at low plasma Ca^2^^+^ through an autocrine mechanism.

## CgA and its cleavage products as biomarkers

Plasma CgA and derived peptides are now commonly used as diagnostic and prognostic markers or to monitor the response to pharmacotherapeutic intervention in several diseases, such as endocrine tumors, heart failure, hypertension, and neurodegenerative and neuropsychiatric diseases (e.g., depression, schizophrenia, and bipolar disease) (50, 51, 52, 53) (Fig. 2B).

### Tumors

NETs represent a heterogeneous family of tumors with different morphological and clinical features originating from a variety of neuroendocrine cell types distributed ubiquitously throughout the body. To date, CgA level, representing a constitutive neuroendocrine secretory protein, is the most widely accepted biomarker, being elevated in 60–80% of patients with NETs (54). Elevated CgA levels correlate with disease burden and poor outcomes (55) and, in pancreatic NETs, an early decline during treatment was associated with improved prognoses (56, 57, 58). However, the utility of serial CgA for monitoring treatment response still remains to be prospectively established (59). Recently, it has also been supposed that CgA is differentially regulated in primary and metastatic small intestinal NETs (60).

### Cardiovascular diseases and hypertension

As CgA is much more stable than catecholamines in the circulatory system, its plasmatic levels reflect the sympathetic tone and adrenomedullary system activity, that are altered in chronic heart failure, acute coronary syndrome, and hypertension. High CgA plasma levels are strictly associated with mortality risk after myocardial infarction or acute coronary syndrome as well as heart failure while increased catestatin concentrations appear to improve post-ischemic recovery by reducing the myocardial infarct size and the increment of diastolic left ventricular pressure (27, 61, 62).

### Inflammatory diseases

Serum CgA has been used as an early biomarker of disease severity in patients admitted with systemic inflammatory response syndrome (63), whereas a relation between TNFα and CgA has been demonstrated in rheumatoid arthritis (64). Stress situations are considered as a significant predisposing factor for immune diseases, and CgA levels have been related to the onset and progression of periodontal diseases.

### Neurological diseases

The potential utility of CgA as a biomarker in neurological disorders has been only recently established. In particular, decreased CgA levels have been detected in the cerebrospinal fluid of canonical, but not late-onset type II Alzheimer's disease, patients (65), and decreased level of vasostatin is characteristically observed in a cohort of patients with Alzheimer's disease compared with those suffering from frontotemporal dementia and healthy controls (66). These data suggest the potential utility of granin fragments in the differential diagnosis of neurodegenerative diseases.

Recently, CgA has been supposed to be a potential biomarker of multiple sclerosis as cerebrospinal fluid from these patients evidenced a significant increase in CgA_194__–__213_ fragment (67).

### Other pathological conditions

Silent atrophic gastritis and gastritis due to *Helicobacter pylori* infection may determine increased CgA levels, as a consequence of chronic elevation in serum gastrin levels (68, 69). In these patients, especially in those treated with proton pump inhibitors, measurement of serum CgA could be useful to monitor hyperplasia of enterochromaffin-like cells of the stomach.

In organ dysfunction such as renal and liver failures, the CgA levels in serum or plasma may also be markedly increased while slightly increased concentrations of CgA have also been observed in ulcerative colitis and Crohn's disease, hyperparathyroidism, hyperthyroidism, and during menopause (probably due to the increased sympathetic tone) and pregnancy (52, 70, 71).

### Measurement of salivary CgA as a biomarker of psychophysical stress

It has recently been reported that CgA is released from human submandibular glands and secreted into saliva (72). Salivary CgA levels are considered as a reliable non-invasive marker of psychological stress (73, 74), such as exposition to situation of anxiety (75, 76, 77) and depressive mood (78, 79). Moreover, salivary CgA changes during the menstrual cycle in women with different degrees of premenstrual psychoemotional symptoms; in particular, a significant late-luteal increase in salivary CgA level was detected, reflecting an increase in sympathetic nerve activity in women experiencing a substantial increase in a cluster of negative psychoemotional symptoms premenstrually (80).

Physical activity is associated with enhanced adrenergic tone. Recent studies have shown that high-intensity exercise significantly increases plasma and salivary CgA levels (81, 82). Moreover, the elevation of salivary CgA levels in basketball players before competition can have a perceived functional effect with respect to the upcoming performance (83).

## CgA sampling and detection

Plasma or serum sampling is broadly used for the laboratory determination of CgA in a wide variety of endocrine and NETs. However, recent studies have analyzed the hypothesis that detection of salivary CgA level may have a higher analytical and diagnostic performance, as salivary sampling is non-invasive, rapid, and, different from the circulating form, CgA in saliva is not bound to other proteins. Even though only few papers are available on this topic, data appear to suggest that, in physiological conditions, circulating and salivary CgA have different routes of secretion: indeed, salivary CgA peaks upon awakening and then quickly decreases to nadir after 1 h and is maintained at a low level throughout the day, whereas plasma CgA did not show any circadian rhythm (84). On the other hand, salivary and plasma concentrations have been found to be correlated in epilepsy cases and in pheochromocytoma (85, 86). These observations suggest that salivary and circulating CgA can be used for clinical application as complementary markers. When salivary CgA is utilized in order to monitor a psychosomatic or physical stress, the sampling time is critical for a correct analysis (82, 83, 87).

## Effects of the *in vivo* administration of CgA and derived peptides

The pleiotropic effects and the pathophysiological implications of CgA and its derived peptides seem to suggest that these molecules bear all the potentials to be therapeutic agents for several diseases. Nevertheless, no clinical trials on the effects of their *in vivo* administration have been registered to date. Experiments performed in genetically modified mice evidenced that catestatin inhibited the nicotine-induced catecholamine secretion, whereas its i.v. administration in rats reduced pressure responses to the sympathetic activation and evoked a potent vasodilation (88). This vasoactive effect has been confirmed in healthy human subjects by infusing catestatin into dorsal hand veins after pharmacological venoconstriction with phenylephrine (38). This vasodilatory effect of catestatin was more important in females, indicating that catestatin may contribute to sex differences in endogenous vascular tone and influence the complex predisposition to hypertension.

## Conclusions

This review summarizes the knowledge about CgA and functions of its cleavage products emphasizing their importance in physiological and pathological conditions. It is worth noting that some of the CgA-derived peptides can exert opposing effects, and therefore, the regulation of the CgA processing to generate diverse molecules under different physiological conditions is critical in order to counterbalance the effects and to maintain homeostasis. The potential use of CgA as a pharmacological agent needs to be investigated to fill the current knowledge gap.

Finally, the application of salivary samples could substitute CgA detection in plasma, for clinical purpose.

## Declaration of interest

The authors declare that there is no conflict of interest that could be perceived as prejudicing the impartiality of the research reported.

## Funding

This paper was supported by PRIN 2010–11 funds of the Italian Ministry of Education, University and Research.
